# Direct reconstruction of chronic extensor digitorum longus tendon rupture using interposed scar tissue in the foot: A case report

**DOI:** 10.1097/MD.0000000000022506

**Published:** 2020-10-02

**Authors:** Eui Dong Yeo, Jong Kyu Han, Hong Seop Lee, Sung Hun Won, Ki Jin Jung, Hee Jun Chang, Joong Suk Cha, Hyein Ahn, Dhong Won Lee, Jin Ku Kang, Woo Jong Kim

**Affiliations:** a Department of Orthopaedic Surgery, Veterans Health Service Medical Center, Seoul; b Department of Radiology, Soonchunhyang University Hospital Cheonan, 31, Suncheonhyang 6-gil, Dongam-gu, Cheonan; c Department of Foot and Ankle Surgery, Nowon Eulji Medical Center, Eulji University, 68, Hangeulbiseok-ro, Nowon-gu, Seoul; d Department of Orthopaedic Surgery, Soonchunhyang University Hospital Seoul, 59, Daesagwan-ro, Yongsan-gu, Seoul; e Department of Orthopedic Surgery, Soonchunhyang University Hospital Cheonan, 31, Suncheonhyang 6-gil; f Department of Pathology, Soonchunhyang University Hospital Cheonan, 31, Suncheonhyang 6-gil, Dongam-gu, Cheonan; g Department of Orthopaedic Surgery, Konkuk University Medical Center, 120-1, Neungdong-ro, Gwangjin-gu, Seoul; h Department of Anesthesiology and Pain Medicine, Soonchunhyang University Hospital Cheonan, 31, Suncheonhyang 6-gil, Dongam-gu, Cheonan, Korea.

**Keywords:** extensor digitorum longus tendon, foot, reconstruction, scar tissue

## Abstract

**Rationale:**

Primary repair of acute ligament injury is possible due to the proximity of the ends. In the case of chronic injury, however, primary repair is difficult because the ends of ruptured ligament will have receded, and tendon graft, transfer, or reconstruction is needed. Satisfactory clinical results have been reported after reconstruction with newly formed interposed scar tissue between the ends of the ruptured tendon in chronic Achilles tendon injury and chronic extensor halluces longus (EHL) tendon injury. Here, we report a patient treated with reconstructive surgery using well-formed scar tissue between the ends in a case of chronic EDL tendon rupture.

**Patient concerns:**

A 34-year-old woman visited the clinic with pain in the dorsum aspect of the right foot associated with weakness and loss of extension of the second toe. She had sustained an injury to the dorsal aspect of her foot by falling on broken glass 3 months before coming to our clinic. The patient reported pain and limitation of the extension of the second toe for 2 months. Her pain continued to worsen, and 1 month later she was transferred to our hospital because a different local clinician suspected she had ruptured her second EDL tendon.

**Diagnosis:**

Magnetic resonance imaging (MRI) revealed complete rupture of the second EDL tendon at the metatarsal neck junction, with displacement of the distal end to the proximal phalanx shaft area and of the proximal end to the metatarsal shaft area.

**Interventions:**

Chronic rupture of the EDL tendon was treated with direct reconstruction using interposed scar tissue.

**Outcomes:**

At the 3-month follow-up, the patient was almost asymptomatic and had nearly full range of motion in dorsiflexion of the second toe. She has no discomfort in her daily life and has returned to almost her preoperative level of functional activities.

**Lessons:**

Here, we presented an extremely rare case of reconstruction using interposed scar tissue in a patient with neglected EDL tendon rupture. Direct reconstruction using interposed scar tissues located between the ends of the ruptured tendon is considered a reliable method with satisfactory clinical results in carefully selected patients.

## 1 Introduction

Laceration of the tendons of the foot and ankle is relatively common.[^1^,^2^,^3^,^4^] Most reports published to date have discussed specific tendon injuries, such as tibialis anterior, extensor halluces longus tendon, Achilles tendon, and flexor halluces longus tendon, and are small case series and technical tips in pathological situations.[^1^,^2^,^3^,^4^,^5^,^6^] Most reported cases of laceration of extensor digitorum longus (EDL) tendons have been treated by primary repair without any specificity, and only a few cases have developed complications.[^1^,^2^,^3^]

In general, primary repair is possible in cases of acute ligament injury due to the proximity of the ends of the ruptured ligament. However, in the case of chronic injury, primary repair is difficult because the ends of ruptured ligament will have receded, and tendon graft, transfer, or reconstruction is needed.^[7]^

Satisfactory clinical results have been reported after reconstruction with newly formed interposed scar tissue between ruptured tendon ends in chronic Achilles tendon injury and chronic extensor halluces longus (EHL) tendon injury.[^5^,^6^,^7^,^8^] Therefore, it was postulated that the above method could be applied in cases of rupture of other tendons.

Here, we report a patient treated with reconstructive surgery using well-formed scar tissue between the ruptured ends of chronic extensor digitorum longus tendon rupture.

## 2 Case description

This case report was approved by the Institutional Review Board of Soonchunhyang University Hospital (IRB No. 2019–12–011). The patient provided written informed consent for publication of this report and the accompanying images.

A 34-year-old woman visited the clinic with pain in the dorsal aspect of the right foot associated with weakness and loss of extension of the second toe. She had sustained an injury to the dorsal aspect of her foot by falling on broken glass 3 months before visiting our clinic. Examination revealed an old scar transversely on the dorsal aspect of the neck portion of the second metatarsal area. According to the patient, only skin closure had been performed at a local clinic at the time of the injury. The patient reported feeling pain corresponding to about 3 points on a visual analog scale (VAS) and limitation of extension of the second toe for 2 months, but felt that this was part of the healing process and did not go to the hospital. Two months after the injury, the patient visited a local clinic where she was diagnosed with no specific findings and was given conservative treatment and physical therapy. The patient was a housewife and there were no signs of rheumatoid arthritis, other systemic diseases, medication use, or history of steroid or other injections in the affected foot. Her pain continued to worsen to 4–5 points on the VAS, and 1 month later she was transferred to our hospital because a different local clinician suspected she had ruptured her second EDL tendon.

**Figure 1:**
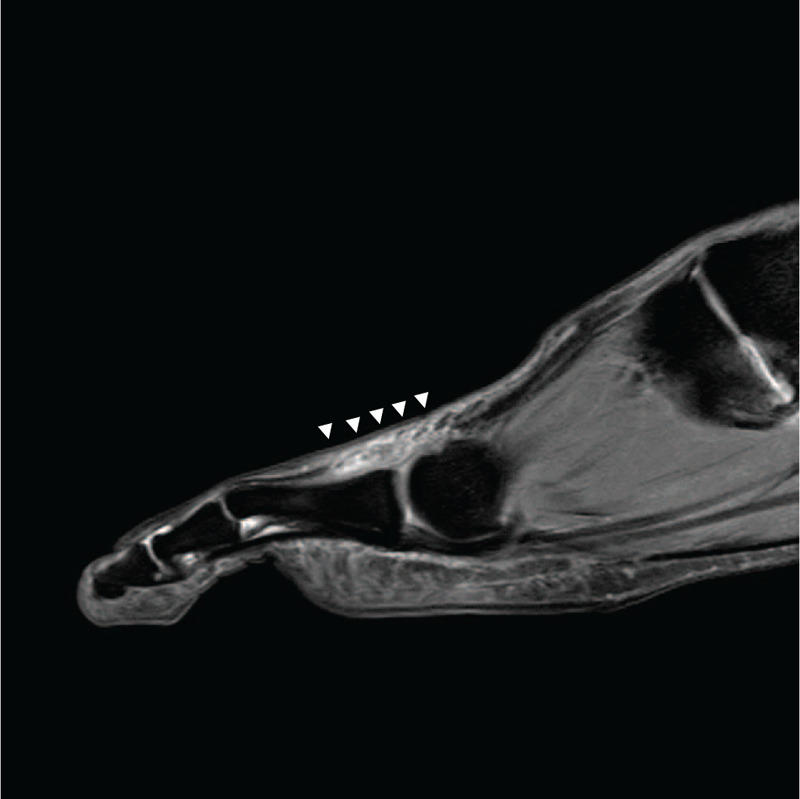
Preoperative T2-weighted sagittal magnetic resonance image showing ruptured second EDL with diffuse intratendinous heterogeneous signal change (white arrowheads).

On physical examination, the patient had mild swelling and tenderness on the dorsal scar area, about 1 cm proximal to the second metatarsophalangeal (MTP) joint, but no palpable mass. Active flexion of the second MTP joint was intact. There was no active extension in the second MTP joint but passive extension was normal. There was no local tenderness at the second interphalangeal (IP) joint. The results of neurovascular examination were unremarkable. She reported nothing more than slight pain in the injured area when she wore shoes, but suffered more pain in the swing phase when barefoot and her second toe was not extended, which caused discomfort. A diagnosis of neglected chronic rupture of the second EDL tendon was made. No specific findings were observed on plain radiography. Magnetic resonance imaging (MRI) revealed complete rupture of the second EDL tendon at the metatarsal neck junction, with the distal end displaced to the proximal phalanx shaft area and the proximal end displaced to the metatarsal shaft area. As a special note, wide and heterogeneous high-signal intensity imaging findings were continuously observed between the ruptured tendon along the EDL tendon in MRI T2-weighted images (Fig. 1). This finding was confirmed at surgery. The patient wanted surgery because the pain and discomfort interfered with her daily life and she wished to recover some movement of the second toe. The patient was presented with various surgical options depending on the surgical findings (tendon graft, transfer, reconstruction), which she understood.

**Figure 2:**
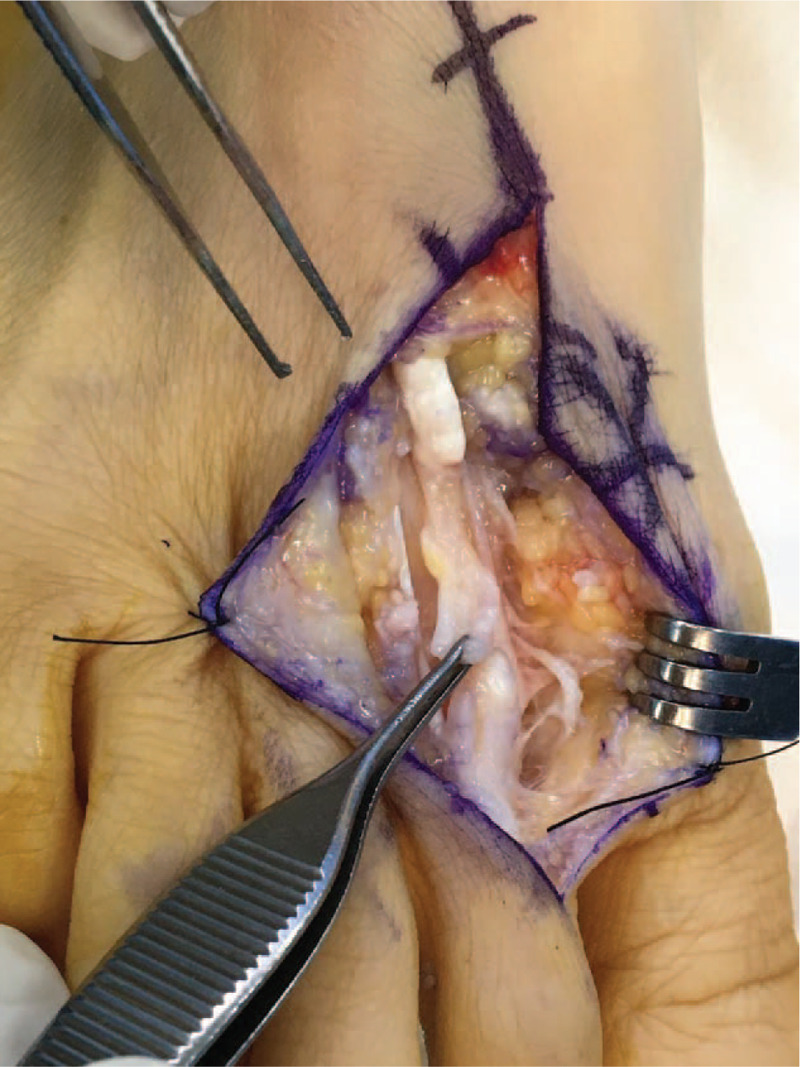
Intraoperative photograph showing rupture of the EDL tendon and interposed scar tissue.

A dorsal longitudinal zig-zag incision was made along the second metatarsal area to expose the site of suspected displacement of the proximal and distal portions of the torn EDL tendon around the scar. When the ruptured site of the EDL tendon was exposed, traces of the ruptured tendon were found but newly formed extensor tendon-like scar tissue was observed uniformly between the rupture ends (Fig. 2). The gap between the ruptures was about 3 cm. The scar tissue was relatively hard and the second toe was pulled to some extent when the scar tissue was pulled. Therefore, we decided to reconstruct the EDL tendon using scar tissue.

**Figure 3:**
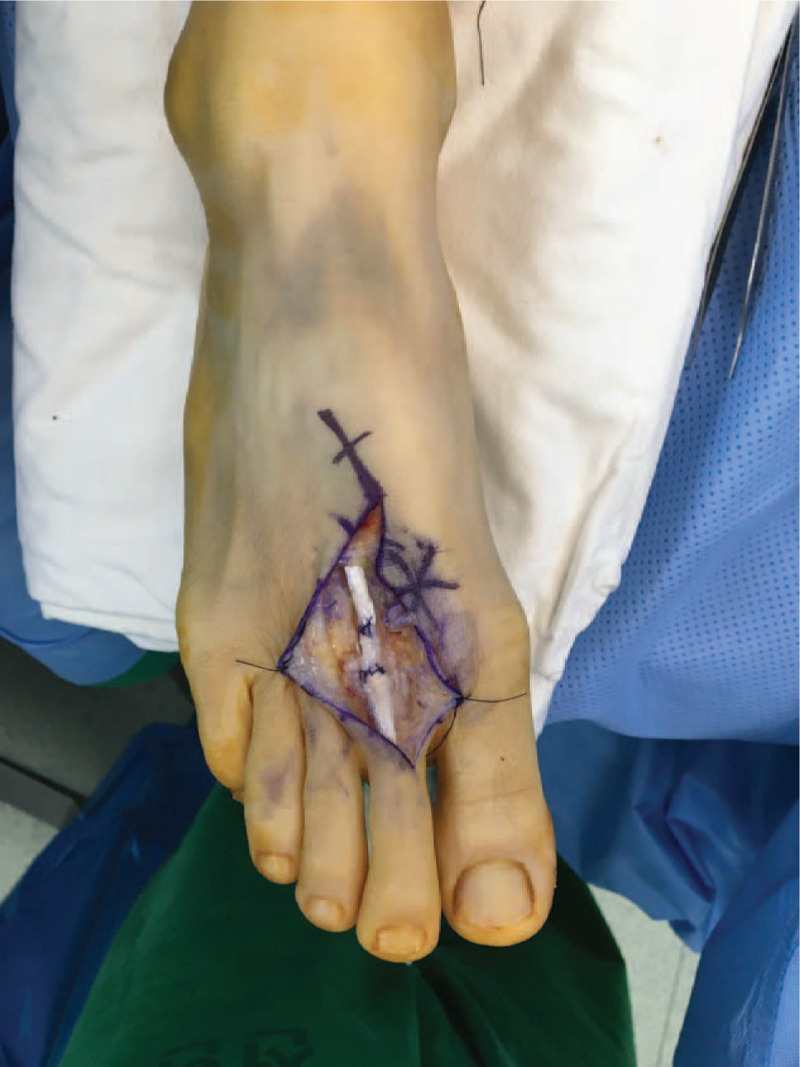
The EDL tendon was reconstructed using interposed scar tissue.

The scar tissue between the ruptures was excised at the border of the proximal rupture, which was weak. The scar tissue was thinner than the normal EDL ligament, so it was folded for reconstruction. Next, the second toe was placed in the neutral position, the scar tissue was folded to the length of the ligament, and both ends and scar tissues were sutured via simple end-to-end suturing (Fig. 3). Some of the excised scar tissue was referred for biopsy.

Postoperatively, the foot and ankle were immobilized for 4 weeks in a short-leg splint, with the ankle and MTP joint in a neutral position. Immediately after the operation, passive MTP joint extension exercise was initiated by manual manipulation but active exercise was not permitted. Active dorsiflexion and progressive passive plantarflexion were performed 6 weeks postoperatively. After 2 weeks, the patient commenced active exercise with slight resistance.

**Figure 4:**
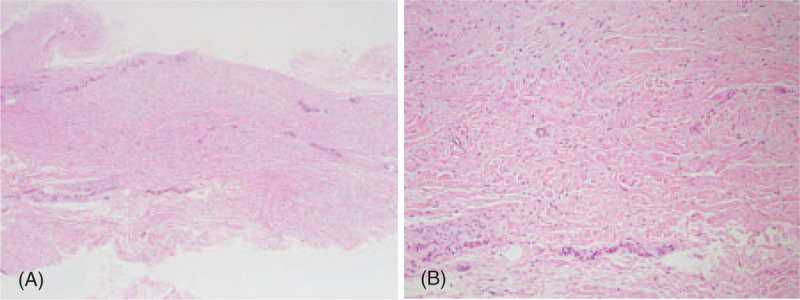
Histological examinations of the interposed scar tissue. (A) Hematoxylin and eosin,×40. (B) Hematoxylin and eosin,×100. Microscopic examination showed fibrous scar tissue composed of thick collagen fibers running parallel to the tendon axis (A) with fibroblasts and mild vascular proliferation (B).

Histological examination of the excised scar tissue between the ruptured ends consisted of dense, thick collagen fibers running parallel along the tendon axis and fibroblasts with vascular proliferation. No degeneration changes in the muscle layer, fat infiltration, or mucoid degeneration were observed (Fig. 4).

**Figure 5:**
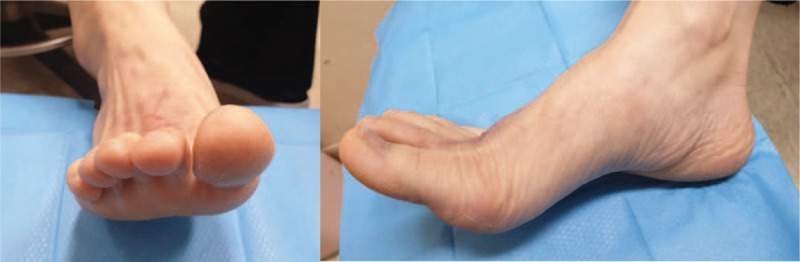
Photographs taken 3 months after surgery showing active range of motion of the operated second toe.

**Figure 6:**
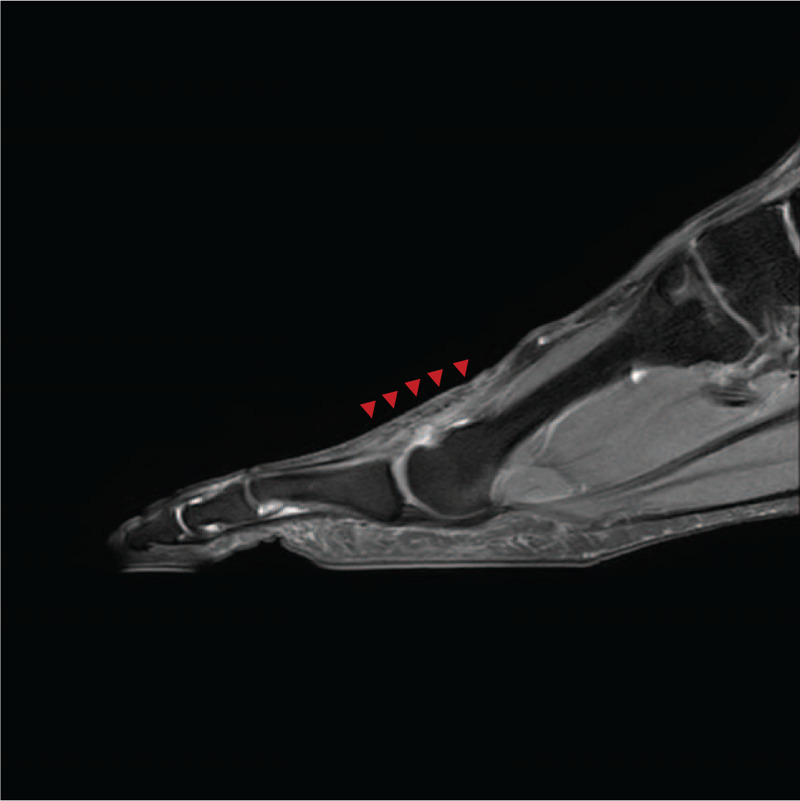
On the proton density-weighted sagittal magnetic resonance image taken 1 year after reconstruction, the second EDL tendon is well maintained. (red arrowheads).

At 3-month follow-up, the patient was almost asymptomatic and had recovered almost full range of motion in dorsiflexion of the second toe (Fig. 5). She has no discomfort in her daily life and has returned to almost her preoperative level of functional activities. At 1-year follow-up, active full dorsiflexion of the second MP joint was possible. Postoperative MRI taken 1 year after reconstruction, the second EDL tendon is well mainted (Fig. 6). She had no complications or recurrent symptoms. The lesser metatarsophalangeal-interphalangeal scale of the American Orthopedic Foot and Ankle Society improved from 69 points preoperatively to 90 points postoperatively, and the VAS score for pain was improved from 4 to 1.

## 3 Discussion

Acute extensor tendon injuries in the foot are common due to their subcutaneous location, and most such injuries are caused by dropping of sharp objects onto the dorsum of the foot.[^3^,^9^]

Thordarson and Christopher^[3]^ reported that isolated laceration of the second to fourth EDL tendons does not cause major problems and suggested that delayed recognition of EDL tendon injuries does not require repair provided the function of the extensor digitorum brevis (EDB) muscles remains intact. Wickes et al^[4]^ also reported that EDL tendon laceration is well tolerated because the adjacent tendon serves as an internal splint. However, in cases of laceration of the fifth EDL tendon, there is no EDB tendon, which can cause flexed toe.

Floyd et al^[2]^ reported complications in unrepaired EDL tendon rupture. They reported recovery of active extension in seven of eight cases of EDL tendon lacerations with primary repair. However, one case with laceration treated only by skin closure presented with moderate, symptomatic, progressive clawing of the fourth and fifth toes of the same foot and no EDL tendon function was present in the second and third toes.

Acute injury of the EDL tendons can be repaired easily if detected early as the tendons are at the subcutaneous level. However, if surgery is needed due to complications caused by the delayed recognition of the injury, a method other than primary repair is required, including tendon graft, tendon transfer, or tendon reconstruction. Although good results have been reported by such reconstructions, they can compromise the normal structure of the donor site and are related to donor site morbidity.^[10]^

In 1997, Porter et al^[11]^ published the results of primary repair using interposed scar tissue in 11 patients with subacute neglected Achilles tendon rupture. They performed overlapping sutures at each end without excision of scar tissue and obtained good functional results. However, this surgery can only be performed if the ends are accessible and primary repair is possible after 20° of plantarflexion. Their operation was not a method of reconstruction, and they suggested that further research was needed to determine whether interposed scar tissue could be used in cases of chronic Achilles tendon rupture.

Interestingly, reconstruction with newly formed interposed scar tissue located between the ruptured tendon stumps has been reported, unlike conventional reconstruction methods for chronic Achilles tendon injury and chronic EHL rupture.[^5^,^6^,^7^,^8^]

Porter et al^[11]^ presented excellent clinical and functional outcomes in patients in the subacute phase between 4 and 12 weeks after Achilles rupture by primary repair without excision of interposed scar tissue. They reported the results of biopsy specimens from interposed fibrovascular scar tissue from 4 to 13 weeks after Achilles tendon rupture. At 4 weeks, residual tendon and reparative processes coexisted, some of which consisted of granulation tissue infiltrated with fibrosis. At 6 weeks, an abundant amount of granulation tissue ingrowth associated with edema was observed, showing a focally obscure tendinous morphology. At 10 weeks, there was focal fragmentation and fibrillation of the tendinous element associated with significant edema indicating more prominent with resultant accentuation of the interspersed tendon fibers. At 13 weeks, a prominent granulation tissue response was observed as proliferation of capillary type vessels adjacent to the tendinous material.

Yasuda et al^[8]^ reported that direct repair methods were effective for healing tendons, including scars without allografts or autografts, in 30 patients with chronic Achilles tendon rupture treated more than 4 weeks after injury. They found connective tissue consisting of dense and thick collagen fibers with vessels in all 30 biopsy specimens in patients with chronic Achilles rupture more than 4 weeks after injury. Degenerative changes, such as tendolipomatosis, mucoid degeneration, necrosis, and vascular changes, were not found in these tissues. In nine cases, dense collagen fibers were found with fibroblasts running parallel to the tendon axis. However, compared to intact tendons, the collagen fibers were thinner and fibroblasts were more abundant. The remaining 21 cases did not show these fibers and fibroblasts running parallel along the tendon axis. Lee and Lee^[5]^ described satisfactory clinical results with reconstruction using interposed scar tissue in a patient with chronic EHL tendon rupture. They found thick collagen fibers running parallel to the tendon axis on biopsy. Similar biopsy results were observed in the interposed scar tissue of our case of chronic EDL rupture.

Several studies have reported preoperative MRI findings as a way to predict preoperative ligament status. Karjalainen et al^[12]^ and Shalabi et al^[13]^ reported that the presentation of a high-signal-intensity area in T2-weighted images in ruptured Achilles tendon represents active scar formation and the healing process. Yasuda et al^[6]^ suggested that for reconstruction using interposed scar tissue, the condition of scar tissue in preoperative T2-weighted MRI should show a thickened fusiform shape with diffuse intratendinous high-signal changes. This method is not indicated if MRI reveals narrowing and focal high-signal changes at the site of rupture. Lee and Lee^[5]^ also described wide and heterogeneous high signal intensities between the stumps following the axis of the EHL tendon in preoperative T2-weighted MRI. Yasuda et al^[8]^ suggested that successful reconstruction could be performed even if thin, elongated scar tissue was seen in preoperative MRI, because resection of the middle of the scar tissue led to fibroblast migration from the stumps. They also assumed that early functional rehabilitation of the suture site may align the bundles of collagen fibers parallel to one another along the axis of the tendon and aid in providing mechanical strength, consistent with previous studies.[^14^,^15^] In our case, resection of the scar tissues to achieve ligament length and tension and early functional rehabilitation yielded satisfactory results.

When considering various reports and literatures,[^8^,^11^,^14^,^15^] as a prerequisite for reconstruction using scar tissue, it is possible from 1 month after tendon injury if scar tissue is well formed on the MRI before surgery. However, the authors believe that the results of the biopsy reported by Porter et al^[11]^ may show good results if reconstruction is performed 3 months after scar tissue matures similar to tendinous material.

## 4 Conclusion

We present an extremely rare case of reconstruction using interposed scar tissue in neglected EDL tendon rupture. Early exploration is important to allow the full definition of the extent of the injury and early surgical repair of tendons is recommended to avoid future disability. The direct reconstruction method using interposed scar tissue located between the ends of the ruptured tendon is considered a reliable method with satisfactory clinical results in carefully selected cases. It is associated with no other donor site damage and has the advantage of simplicity over other surgical methods. Further studies are required to determine whether this method is feasible for ligament injury at other sites.

## Author contributions

**Conceptualization:** Eui Dong Yeo, Woo Jong Kim.

**Data curation:** Jong Kyu Han.

**Investigation:** Hee Jun Chang, Joong Suk Cha, Woo Jong Kim.

**Methodology:** Sung Hun Won, Hyein Ahn.

**Project administration:** Hee Jun Chang.

**Resources:** Hyein Ahn.

**Software:** Ki Jin Jung, Jin Ku Kang, Woo Jong Kim.

**Supervision:** Jong Kyu Han, Sung Hun Won, Woo Jong Kim.

**Visualization:** Hong Seop Lee, Hee Jun Chang, Hyein Ahn, Dhong Won Lee, Woo Jong Kim.

**Writing – original draft:** Woo Jong Kim.

**Writing – review & editing:** Woo Jong Kim.
